# Transactions of the Medical Society of the County of Kings

**Published:** 1865-11

**Authors:** Wm. C. Otterson

**Affiliations:** Late Surgeon U. S. Volunteers


VOL. V. NOVEMBER, 1865. No. 4.

ART. I.—*Transactions of the Medical Society of the County of Kings.*

### NOTES ON MILITARY SURGERY.

BY WM. C. OTTERSON, LATE SURGEON U. S. VOLUNTEERS.

REGULAR MEETING, JULY, 1865.

In making the promise to present to the Society a paper on "Military Surgery," I had in view a brief synopsis of some of my most vivid recollections of army experience. In the scope of a communication of this character but very small portions of so wide and fertile a field as that of Military Surgery can be included, and I shall confine myself to the consideration of the changes wrought in the constitution of the volunteer soldier by the new influences to which he is subjected. A few remarks on the frequency, character and treatment of the wounds produced by weapons of war, and a notice of some of the diseases peculiar to large military hospitals.

The raw recruit, as he falls under the eye of the surgeon, differs in some respects from patients of any other class. The radical change from a life of peace to one of war produces no less change in the physical than in the moral constitution. The farmer, the mechanic, the tradesman from the country, has been accustomed to quiet, regular habits, to good food, well prepared, to good beds and shelter, and to clothing sufficient for cleanliness and comfort. Often those from the city are broken down by dissipation and excesses. In the soldier's life the food is often insufficient and almost always badly prepared, there is exposure to the vicissitudes of season and climate with either no shelter, or what is worse, the shelter of crowded, illy-ventilated barracks, reeking with the mephitic exhalations of organic excrementitious matters. The frequent recurrence, and long continuance of those pestiferous influences in railroad cars and transports thoroughly poisons the system, and even before the soldier reaches the field he bears, implanted within him the fertile seeds of disease, or is dropped by the way-side, rarely to rejoin his command. Acute scorbutus, acute diarrhœa and dysentery, typhoid fever, erysipelas, pneumonia now prevail, and not so in civil practice. We are often utterly powerless to remove the exciting causes. The veteran who has passed safely through this stage of the soldier's life, bears his ills with greater endurance, we cannot say indifference, for no man can be indifferent to his own sufferings; but there is no pang more intense than that of the young recruit when he first falls sick, and fully realizes that he, among strangers, away from home—a home, though poor it may be, whose associations are all intensified by his nostalic condition. His physical infirmity may be mild, but if sufficient to separate him from his companions, his mental suffering is intensified by his powerless confinement and subordination. Coleridge says—

> "Home-sickness is a wasting pang,
> This feel I hourly, more and more;
> There's healing *only* in thy wings,
> Thou breeze that play'st on Albion's shore."

If the deleterious influences I have mentioned are so productive of disease, how much more pernicious must they be when acting on the system of a sick or wounded man? Must these evils remain the inevitable consequences of war, or will sanitary science step in to mitigate or remove them? When will the practical fact be duly recognized that the prevention of disease is vastly more economical of life and treasure than its cure?

I can only glance at a few of the diseases met with by the military surgeon in camp and hospital in the scope of such a paper as this, but of these I will choose the most prominent and distinctive.

*Erysipelatous Pneumonia.*—Those liable to this disease may be of any age or temperament, the oldest and most infirm being, of course, most subject to its attack. The patient may be admitted to the hospital with any of the diseases or wounds incident to a soldier's life. In a few days pneumonia is developed, and soon after (two or three days) erysipelas sets in, which may be either idiopathic or traumatic. If the former, it is most apt to attack the head, face or chest, but will often occur on the extremities. The erysipelas may even precede the pneumonia, the latter following the sudden disappearance or metastasis of the former. In such cases I have frequently called the attention of the medical staff of the hospital to patients where erysipelas had suddenly disappeared, predicting the development of pneumonia within forty-eight hours, the prognosis being rarely falsified by the result. I am well satisfied that this occurs too often to be a mere coincidence, and that the same influences and *materies morbi* may produce either erysipelas or pneumonia, or both; that where erysipelas prevails in large hospitals, as an epidemic, the number of cases of pneumonia is *always* greatly increased. I do not mean to say that *every* case of pneumonia will be attended with or followed by erysipelas, or conversely; the observation applies to those cases in which one or the other of these diseases is developed in the wards of a large hospital. The treatment should always be that of tonics and stimulants, with the most nutritious diet. The mortality is always large, and the post-mortem changes are found mainly in the lungs and brain.

*Wounds.*—By far the least frequent are those inflicted with the bayonet, as charges *en masse* are hardly ever received by the party assailed. The saber wound is also rare. We often read of cavalry charges, but seldom see on the field or in the hospital the terrible consequences we would be led to expect. In the charge of the "light brigade" the slaughter was produced by "cannon to right of them, cannon to left of them," and was inflicted *upon* the cavalry. A hand-to-hand conflict between cavalry, with the use of the saber, is very rare in our mode of fighting, and it is seldom that in flanking infantry do not escape without being badly cut up or surrender without much resistance. Our cavalry is armed with carbine and pistol, on which they rely rather than on the saber.

Next in frequency are wounds from batteries, from round-shot, shell or shrapnel; but by far the most frequent are those from small arms, rifle, musket and pistol. The penetrating wound from a bayonet is grave, according to its situation and depth; the saber wound combines the incised, lacerated and contused. Wounds from round shot and shell are almost always fatal if there is a breach of the integument; for the most part they are lacerated; in those from the explosion of shell the laceration of muscle and the comminution of bone are frightful; the part looks as if it had passed through a threshing-machine; in wounds from round shot and shell death is most often instantaneous.

The conical rifle ball produces a very ugly wound, lacerating the soft parts, and in coming in contact with a bone (if not spent) comminuting it extensively. In striking the shaft of a long bone the splitting for several inches is as if done with a dull hatchet; the round ball from the old fashioned musket is far less destructive, and it is often diverted from the direction by contact with bone.

*Treatment of Wounds.*—A wound inflicted by a weapon of war, *never* heals by first intention; the suture and adhesive strap are seldom needed, and if used, almost always do harm. Foreign bodies in wounds should be removed without delay, if accessible, but persistent probing and cutting for balls, etc., is *meddlesome surgery* and bad practice. If the foreign body lies near a joint or a vital organ, and can be detected there, it should be removed at all hazards. Under ordinary circumstances the finger is the best probe, but when the wound is small or the track deep Nélaton's porcelain-capped probe will often decide a doubtful point. All gun-shot wounds should be dressed for the first few days with lint wetted with cold water. No other local treatment is required. No constitutional treatment is usually indicated; care must be taken not to depress the vital powers; their sustenance is oftener demanded. Unless the missile divides a large artery, and the patient dies from loss of blood before he can be removed from the field, hemorrhage is not so great nor so fatal as would be supposed; the laceration of the artery and of the tissues is so great that the patient faints from shock and loss of blood and the flow ceases. If by the sloughing of a wound of the extremities a principal or secondary artery become involved and secondary hemorrhage suddenly occurs, carefully adjusted compression with the moderate use of the tourniquet should first be used, but the persistent tightening of compress and bandage, if the hemorrhage returns is not, according to my observation, good practice.—

Neither will the ligation of the principal trunk save your patient or his limb. Amputation in these cases is the only safe and sound practice. Where wounds of arteries occur in the neck we have no choice but ligation; both of the artery and vein it may be; if possible on both sides of the wound in the artery.

Of wounds of the cavities, the head, chest, abdomen and bladder a considerable proportion will demand the care and skill of the surgeon. All foreign bodies should be removed; a fragment of bone is as much a foreign body as a piece of shell, wood or cloth, or a bullet, or grape-shot. It must be borne in mind that very extensive wounds of the cavities are not necessarily fatal. Cases of recovery are on record where nearly half the head has been blown off, and I have seen quite a number of cases recover where a grape-shot or Minié ball had traversed the chest, abdomen or bladder. In wounds of the skull the trephine is rarely required, as depressed bone can almost always be elevated through the wound produced by the missile. The head should be shaved; if there is hernia cerebri with laceration of the meninges, that portion which cannot be reduced should be smoothly shaved off with a sharp knife. A compress and light roller kept constantly wet with water completes the local dressing. If the patient can swallow he should be freely stimulated and nourished with food easily elaborated. Opium may be given if necessary without that fear which has always attached to its use in injuries and diseases of the brain. Ice to the head and stimulating injections fulfill important indications. If the bladder is paralyzed, the catheter should be used once in twelve hours; this should be carefully looked after in all severe injuries where the patient is unconscious.

In wounds of the chest there is little to be done locally. I have had no experience in "hermetically sealing" these wounds, and think that while it might occasionally act well, in most cases it would be decidedly injurious. It is no doubt owing to the collapse of the lung when wounded that so many recoveries are on record. The patient should be placed in the most comfortable position, which will usually be the sitting position, giving the thoracic and abdominal muscles full play in the movements of respiration. Bandaging the chest in these cases tends to further embarrass the breathing, and hence should be discarded. A few thicknesses of wet lint over the wound is all that is required.

In wounds of the stomach or abdomen keep clean, give full doses of opium; nourishment and stimulants may be given by the mouth or per anum, as the case may demand. Where the intestines are wounded and protrude, they should be carefully washed with warm water; should be sewed up by the glover's stitch, with iron or silver wire, and returned; the wound should be closed with adhesive straps, and a broad bandage fastened around the abdomen.

Where the bladder has been wounded, so as to give rise to extravasation of urine, the catheter should be retained in the bladder either through the wound or through the urethra; but if its presence give rise to great uneasiness and nervous irritability, it should be removed at intervals to avoid more serious symptoms from its retention than from the passage of urine through the track of the wound. A ball or other foreign body lodging in the bladder may sometimes be removed by simply enlarging the wound or by the operation of lithotomy.

Wounds of the penis, when they involve the track of the urethra, are sometimes quite troublesome, and without great care the contraction of cicatrization produces not only deformity of the organ, but almost entire occlusion of the urethra. The same rule obtains here with regard to keeping the catheter in situ as in wounds of the bladder. When the process of cicatrization begins a stiff leather splint, properly moulded and fenestrated to allow the escape of discharges will sometimes be useful in preventing deformity. Extensive sloughing and infiltration will frequently occur; the first must be met by active escharotics, such as nitric acid, permanganate of potassa, bromine, etc., the second by free incisions made in the long diameter of the organ. Milder cases of sloughing may be counteracted by the application of chlorine water, charcoal, beer, etc. The constitutional treatment must be stimulating and anodyne, with nutritious diet.

Wounds of the scrotum, involving the testes, and producing hernia of the tubuli semeniferi should be treated by extirpation of the wounded organ, as under other circumstances the healing process is very tedious, and the function of the organ destroyed. Where the operation is resorted to promptly recovery is usually rapid.

Probably no class of cases causes the military surgeon so much anxiety, or is the subject of such various opinion and practice as compound fractures of the femur. Unfortunately in a large number of cases the *law* of *expediency* must overrule all other considerations. However much opposed the surgeon may be to immediate amputation, as a rule, its propriety ceases to be a question when the patient must be transported many miles over execrable roads, before reaching the shelter of a hospital. I trust the plaster of Paris splint, as recommended by Dr. James Little, of New York, may be thoroughly tested, and hope it may overcome the painful difficulties of transporting cases of fracture. Many lives and limbs might be saved could the patient be transported without jarring and laceration and grinding of the tissues by the fragments of broken bone. The great majority of our best military surgeons are in favor of the conservative plan of treatment. The course I invariably adopted, and it met the approbation of my medical staff, was to give the patient the benefit of the doubt when there was any chance of saving the limb; never to make a *primary* thigh amputation where the chances that *life* (not the limb) could be saved by putting off the operation. It must be borne in mind that we often attempt to save a limb with little hope of its being ultimately useful where immediate amputation would almost surely prove fatal. After the battles of Chickamauga and Lookout Mountain, where the brave Eleventh, with Hooker at its head, bore our victorious banner above the clouds, a great many compound fractures of the femur came under my observation. At the end of three months most of these cases were doing well, and in quite a number there was a fair prospect of a moderately useful limb. It may be asked what kind of apparatus is best suited for cases. Machinery for fractured femur is as varied as the surgeons who may have to use it are numerous. The surgeon should bear in mind that his first care must be to save the patient's life; his second to save the patient's limb; his third to make him as good and useful a limb after the critical stage has been passed as the patient's constitution and his own skill will permit. For the rational treatment of compound fractures of the femur by gunshot wounds, it is quite impossible to lay down any but general rules, as almost every case requires a method adapted to its own peculiarities. After removing any loose spiculæ of bone and foreign matters, put the patient in the most comfortable position with sufficient dressings to steady the limb and control muscular action, and take care of the general system. To persist in the methods of Liston, Desault, Physic or Smith, will often be to lose your patient in trying to give him a good limb. In the early stages extension and counter-extension can be borne in but few cases. The many-tailed bandage to quiet muscular contractions; a few strips of paste-board, cushions to keep the limb from rolling, perhaps a long side splint, these are all that can be used with advantage during the first month; after this, if the patient is in a proper condition, an apparatns may be applied more with a view to steadiness and straightness than with the hope of securing the required length of limb, as only the most moderate extension will be tolerated. Muscular action will almost always cause angular deformity, and there is no plan of treatment, that I know of, yet devised to overcome this difficulty. The flaccidity of the large muscles of the thigh by long continued pressure may be relieved by Smith's anterior splint, or some of its modifications, if not contra indicated by other considerations. When the bony union is sufficiently firm and suppuration not to profuse, the application of the starched bandage, nicely adjusted and fenestrated if necessary, will produce favorable results. When this has become firm, if the patient has sufficient strength, he may be given a pair of crutches, have the foot suspended in a sling and be allowed to go about. There is one more point in the treatment of these cases of which I wish to speak. For many months after such an injury as has been described, there will be fistulous openings and sinuses indicating the presence of dead bone, and if a probe is introduced it comes into contact with sequestra in almost all directions. What is the best course to pursue in such cases? I answer unhesitatingly, *let them alone.* Any explorative operation will, in a great majority of cases, lead the surgeon upon shoals and quicksands on which he or his patient will be surely wrecked. The new bony material is thrown out in such large quantities and is so intimately interlaced with the splintered shaft, that to attempt the removal of dead bone by forceps, saw, and gouge, will endanger the breaking up of all boney union and leave the patient in a worse condition than when he first fell; may render amputation imperative and sacrifice the life of the patient. Mild, astringent and stimulating injections may be necessary to moderate the discharge and conduce to cleanliness, but no further local treatment is indicated. The general condition of the patient should be carefully watched and his vital resources husbanded. Pain should be quieted by opium, fever allayed by cooling drinks and saline mixtures, the body should be sponged with alcohol and water; the flagging energies of life stimulated and sustained. Where there is a lack of bony material in the system the phosphates of lime and soda may be used with decided advantage. The dangerous complications that arise are irritative fever, erysipelas, gangrene, pyæmia, exhaustive suppuration, secondary hemorrhage, etc. These are the lions in the way and unfortunately for the poor patient are ever unchained. The surgeon must stand as a wary sentinel to challenge all these enemies, to repel them with vigor, and if possible, put them to flight. The treatment of these complications opens too wide a field for present discussion. I will limit myself to a few words on that scourge of military hospitals, *hospital gangrene.*

The treatment of this disease has been made the subject of a monograph by Dr. Goldsmith of Louisville, Kentucky, in which he claims to be the author of the bromine treatment. This opinion was endorsed by Dr. Goldsmith's friends, Dr. Hammond, ex-Surgeon-General, and Dr. Wood, Assistant Surgeon-General, who recommended that the book and bromine should be added to the supply-table. I have no fault to find with the use of bromine as an adjuvant in this disease, but I do most decidedly objeet to an unfounded claim of pre-eminence and originality. As early as 1826 chloride of bromine entered largely into the composition of a salve or paste that was used as an escharotic in malignant ulcerations, and was brought into disrepute by the claims of quacks for the cure of cancer. Any one who will take the trouble to consult the Prussian Pharmacopæia will find that the idea thus given has been simply elaborated by Dr. Goldsmith. Moreover it is now quite generally conceded that bromine does *not* possess any specific influence over hospital gangrene, nor any special advantage over nitric acid or permaganate of potassa, and that it is the most painful of all escharotics. Hospital gangrene has long been recognized as a local disease, yet Dr. Goldsmith considers himself

"very bold," (I use his own language) when he claims this as a new doctrine. It is highly communicable from one patient to another by basons, sponges, the agency of insects, etc. The first indication for its treatment is the removal of the decaying animal tissue as rapidly as disintegration takes place so as to prevent the infection of the sound tissue beneath the diseased. When a stump is attacked after amputation or the disease attacks a wound of the extremities the tepid water douche is one of the most efficient and comfortable applications. A most simple and useful contrivance for its administration is a tin vessel with the spout inverted like an old fashioned watering-pot; to the water may be added a solution of chlorine, tincture of bark or any gentle stimulant or disinfectant. When the gangrene occupies the track of a deep and tortuous wound the frequent injection of such solutions will be highly beneficial. Should the disease continue to extend after removing thoroughly all accessible dead and sloughing tissue make a free and thorough application of some escharotic; strong nitric acid is about as convenient, and its results quite as satisfactory, as any I have seen employed.

In comparing the mortality from hospital gangrene in our own army and that of the English army in the Crimea, it is seneless to attribute to any one remedy a difference for which there were so many adequate causes. The English army was stationary; where they first pitched their tents there they were compelled to remain till the close of the siege. The army of the United States has been an army of moving columns. The English army was poorly supplied with shelter, commissary and quarter-master's stores through a long winter, in a proverbially unhealthy climate, without fuel, except the few roots they could dig after a day or a night in the trenches, through rain, cold and mud; their scanty meal was thus prepared by the *smoke* they could extract from the smouldering of wet roots. Camp diarrhœa, dysentery and scorbutus were their scourges, and the hospitals were filled with cases of gangrene. The sick and wounded of the English army were thus huddled together in illy ventilated and filthy transports, and sent to hospital across the Black Sea. It is no wonder that men subjected to such influences died of hospital gangrene. A summary of the treatment of hospital gangrene would be, 1st, the observance of all hygienic laws; 2d, full stimulation and support of the vital forces; 3d, the local application of detergents and disinfectants; 4th, cauterization by nitric acid, permanganate of potassa, bromine or the actual cautery.

